# Characterization of adipose-derived stem cells from subcutaneous and visceral adipose tissues and their function in breast cancer cells

**DOI:** 10.18632/oncotarget.5922

**Published:** 2015-09-30

**Authors:** Andreas Ritter, Alexandra Friemel, Friderike Fornoff, Mouhib Adjan, Christine Solbach, Juping Yuan, Frank Louwen

**Affiliations:** ^1^ Department of Gynecology and Obstetrics, School of Medicine, J. W. Goethe-University, Frankfurt, Germany

**Keywords:** adipose-derived stem cells, breast cancer cells, epithelial-to-mesenchymal transition, invasion, drug resistance

## Abstract

Adipose-derived stem cells are capable of differentiating into multiple cell types and thus considered useful for regenerative medicine. However, this differentiation feature seems to be associated with tumor initiation and metastasis raising safety concerns, which requires further investigation. In this study, we isolated adipose-derived stem cells from subcutaneous as well as from visceral adipose tissues of the same donor and systematically compared their features. Although being characteristic of mesenchymal stem cells, subcutaneous adipose-derived stem cells tend to be spindle form-like and are more able to home to cancer cells, whereas visceral adipose-derived stem cells incline to be “epithelial”-like and more competent to differentiate. Moreover, compared to subcutaneous adipose-derived stem cells, visceral adipose-derived stem cells are more capable of promoting proliferation, inducing the epithelial-to-mesenchymal transition, enhancing migration and invasion of breast cancer cells by cell-cell contact and by secreting interleukins such as IL-6 and IL-8. Importantly, ASCs affect the low malignant breast cancer cells MCF-7 more than the highly metastatic MDA-MB-231 cells. Induction of the epithelial-to-mesenchymal transition is mediated by the activation of multiple pathways especially the PI3K/AKT signaling in breast cancer cells. BCL6, an important player in B-cell lymphoma and breast cancer progression, is crucial for this transition. Finally, this transition fuels malignant properties of breast cancer cells and render them resistant to ATP competitive Polo-like kinase 1 inhibitors BI 2535 and BI 6727.

## INTRODUCTION

Breast cancer is the second most common cancer worldwide with 1.7 million cases and over 522,000 deaths per year [1]. During the last years, numerous investigations have reported a complex signaling network between breast cancer cells and the tumor microenvironment which affects tumor malignance and therapeutic responsiveness [2]. Adipose tissue, a metabolically active endocrine organ, is the most abundant stromal constituent in the mammary gland surrounding breast cancer. This tissue shows a high secretive activity and is an extensive source of mesenchymal stem cells (MSCs) or more specifically adipose-derived stem/stroma cells (ASCs). ASCs are capable of differentiating into multiple cell types such as adipocytes, neurons, osteoblasts and retain the ability of self-renewing [3]. By virtue of their regenerative features like pro-angiogenic, anti-apoptotic, pro-proliferative and multipotent differentiation, ASCs are widely considered as a promising tool for regenerative medicine application and tissue engineering [4, 5], such as breast reconstruction after breast ablation in cancer patients. Unfortunately, the same features of ASCs that induce tissue regeneration and vascularization are also assumed to be associated with tumor initiation and metastasis [6] raising safety concerns about clinical utilization. Therefore, ASCs have been intensively investigated for their role in cancer cell proliferation, migration and metastasis [7]. While numerous investigations indicate that ASCs promote tumor growth and malignant phenotypes in multiple cancer types [7], supported by *in vivo* studies showing increased tumor growth, metastatic spread and angiogenesis [7, 8], other studies reveal a therapeutic potential of ASCs in breast cancer models *in vitro* and *in vivo* [9, 10].

To further delineate the relationship between ASCs and cancer progression, we have isolated ASCs from visceral and subcutaneous adipose tissue collected from female donors undergoing caesarian section, characterized their features and studied their impact on breast cancer cells. To exclude variations between isolated ASCs from different donors, we performed most of the studies with paired visceral and subcutaneous ASCs of the same donor with a comprehensive number. Our study reveals distinct properties of these two types of ASCs with varied effects on cancer cells. Interestingly, visceral ASCs are more potent to induce the epithelial-to-mesenchymal transition in breast cancer cells mediated by activating multiple pathways in particular the PI3K/AKT signaling.

## RESULTS

### Visceral and subcutaneous ASCs display distinct morphologies and multipotent differentiation potential

**Figure 1:**
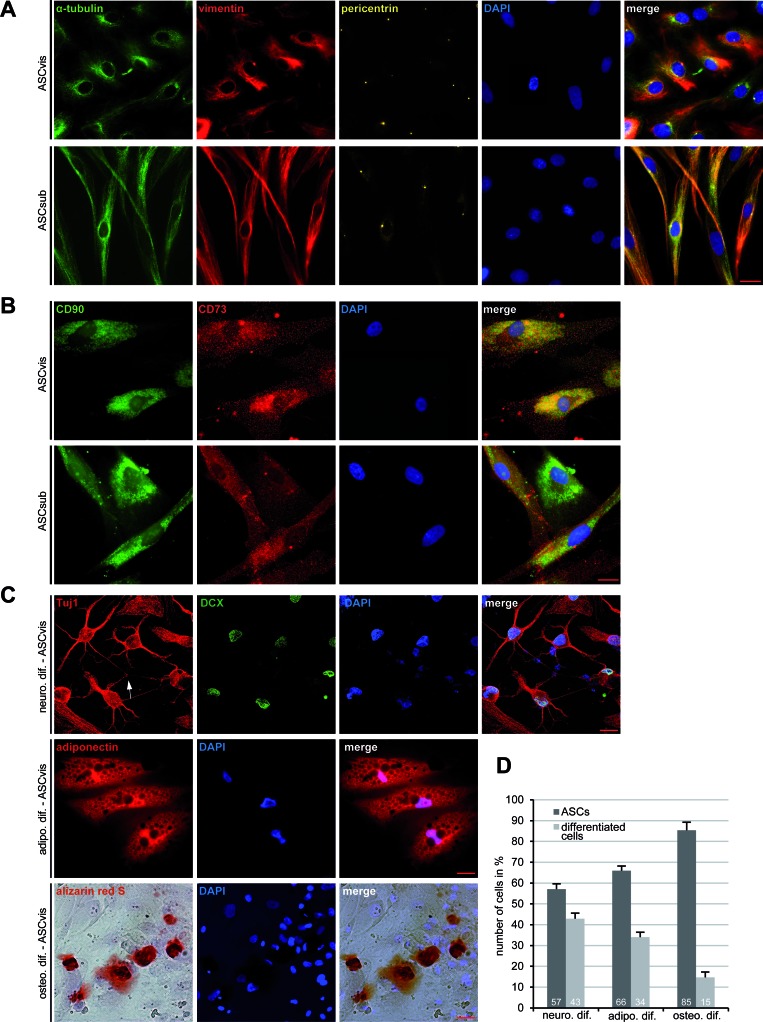
Morphology and differentiation of ASCs isolated from subcutaneous and visceral adipose tissue **A.** Immunofluorescence staining. Visceral ASCs (ASCvis) and subcutaneous ASCs (ASCsub) were fixed and stained for α-tubulin, vimentin, pericentrin and DNA. Examples are shown. Scale bar: 20 μm. **B.** Immunofluorescence staining of cell surface markers in ASCs. Scale bar: 10 μm. **C.** Multilineage differentiation capability of ASCs. ASCs were incubated with corresponding differentiation medium for 14 or 21 days. Neuronal differentiation (14 days) was verified with lineage-specific staining of Tuj1 and DCX. Adipogenic differentiation (14 days) was assessed by oil red O staining displaying lipid vesicle formation. Osteogenic differentiation (21 days) was examined by Alizarin Red S staining indicating calcium deposits. **D.** Quantification of the differentiation potential of visceral ASCs by analyzing lineage-specific characteristic marker (*n* = 300 cells for each condition). The results are based on three independent experiments with ASCs obtained from three different donors and presented as mean ± SEM (*n* = 3).

ASCs were isolated from visceral and subcutaneous adipose tissues, using a well-established method [11], from female donors undergoing caesarian sections (Table 1). These two types of ASCs displayed distinct morphologies at their early passages 1-3: visceral ASCs were more “epithelial”-like with an apical-basal polarity of the tubulin and vimentin cytoskeleton (Figure 1A, 1^st^ panel), whereas subcutaneous ASCs were more characteristic of a fibroblast-like morphology with a small cell body (Figure 1A, 2^nd^ panel). Yet, ASCs isolated from both sources exhibited typical cell surface markers for mesenchymal stem cells described by the Society for Cellular Therapy [11, 12]: positive for CD90, CD73, CD146 and highly negative for CD14, CD31, CD106 and CD34 measured by flow cytometry (Table 2). Indirect immunofluorescence staining in ASCs further underscored the positive signals of CD90 and CD73 (Figure 1B), which were negative in MCF-7 cells (Figure S1A). In addition, the signals of CD14 and CD31 were undetectable in ASCs using immunofluorescence staining (Figure S1B). ASCs were then induced into adipogenic, neurogenic and osteogenic cells, and the *in vitro* differentiation potential was determined by lineage-specific staining. After 14 days of neurogenic induction, 43% of visceral ASCs showed lineage specific staining of Tuj1, a marker for class III β-tubulin, and DCX, a marker for developing neurons, in addition to neuronal branching among differentiated cells (Figure 1C, 1^st^ panel and Figure 1D). 34% of visceral ASCs were positively stained for adiponectin, one of the adipokines secreted by adipocytes, confirming the adipogenic differentiation capacity (Figure 1C, 2^nd^ panel and Figure 1D). The osteogenic differentiation was verified by alizarin red S staining in 15% of cells (Figure 1C, 3^rd^ panel and Figure 1D). All these differentiation markers were negative in non-differentiated ASCs (Figure S1C). Moreover, compared to visceral ASCs, subcutaneous ASCs of the same donor displayed less differentiating capability by showing only 37% positive in neuronal markers, 29% in adipogenic markers and 9% in alizarin red S (Figure S1D), indicating that these two types of ASCs exhibit not only varied morphology but also different differential potential.

**Table 1:** Clinical information of 10 patients

	age	gestational age (weeks)	body mass index (BMI)	birth weight (g)
patients	33.6 ± 3.3	38.2 ± 2.6	22.0 ± 2.5	3295 ± 666

**Table 2:** Cell surface markers of ASCs

	Cell surface markers in %						
Patient No.:	CD90	CD73	CD146	CD14	CD31	CD106	CD34
No. 1: ASCvis	62.88	45.58	8.46	6.90	0.61	0.84	ND.
ASCsub	76.92	90.13	18.84	7.20	2.03	1.63	ND.
No. 2: ASCvis	96.39	92.04	18.33	8.57	2.50	2.14	ND.
ASCsub	75.39	94.63	23.07	0.20	1.11	1.04	ND.
No. 3: ASCvis	62.14	75.89	49.28	2.02	2.55	2.08	ND.
ASCsub	58.65	94.00	49.75	0.50	2.60	2.12	ND.
No. 4: ASCvis	81.84	55.17	3.89	16.77	2.49	0.23	1.97
ASCsub	81.36	74.52	6.07	10.54	2.44	1.39	2.64
No. 5: ASCvis	64.69	97.37	79.33	0.67	2.88	6.84	0.18
ASCsub	88.72	64.34	71.72	3.22	0.64	0.54	4.36
No. 6: ASCvis	38.47	58.02	50.43	11.94	1.51	5.94	0.04
ASCsub	97.67	91.37	75.24	1.47	2.60	5.73	0.17
No. 7: ASCvis	91.60	33.09	18.94	1.05	1.99	5.14	1.07
ASCsub	47.47	87.99	30.45	7.95	1.85	4.94	0.89
No. 8: ASCvis	89.66	94.01	84.59	2.60	2.02	1.86	ND.
ASCsub	87.44	91.58	93.28	19.94	2.54	2.09	ND.
No. 9: ASCvis	79.36	92.40	20.39	0.41	1.93	1.92	ND.
ASCsub	9.86	94.31	29.58	0.13	1.70	9.45	ND.
No. 10: ASCvis	80.42	72.11	58.96	5.93	0.95	1.78	ND.
ASCsub	71.87	98.51	65.98	8.04	2.51	2.05	ND.
Mean value:	72.14	79.85	42.83	5.80	1.97	2.99	1.41
SD value:	21.54	19.23	28.53	5.71	0.69	2.47	1.50

Values represent the percentage of ASCs expressing different surface specific markers.

ASCvis, visceral adipose-derived stem cells; ASCsub, subcutaneous adipose-derived stem cells,

ND., not determined.

### ASCs secrete various factors and are attracted to breast cancer cells

**Figure 2:**
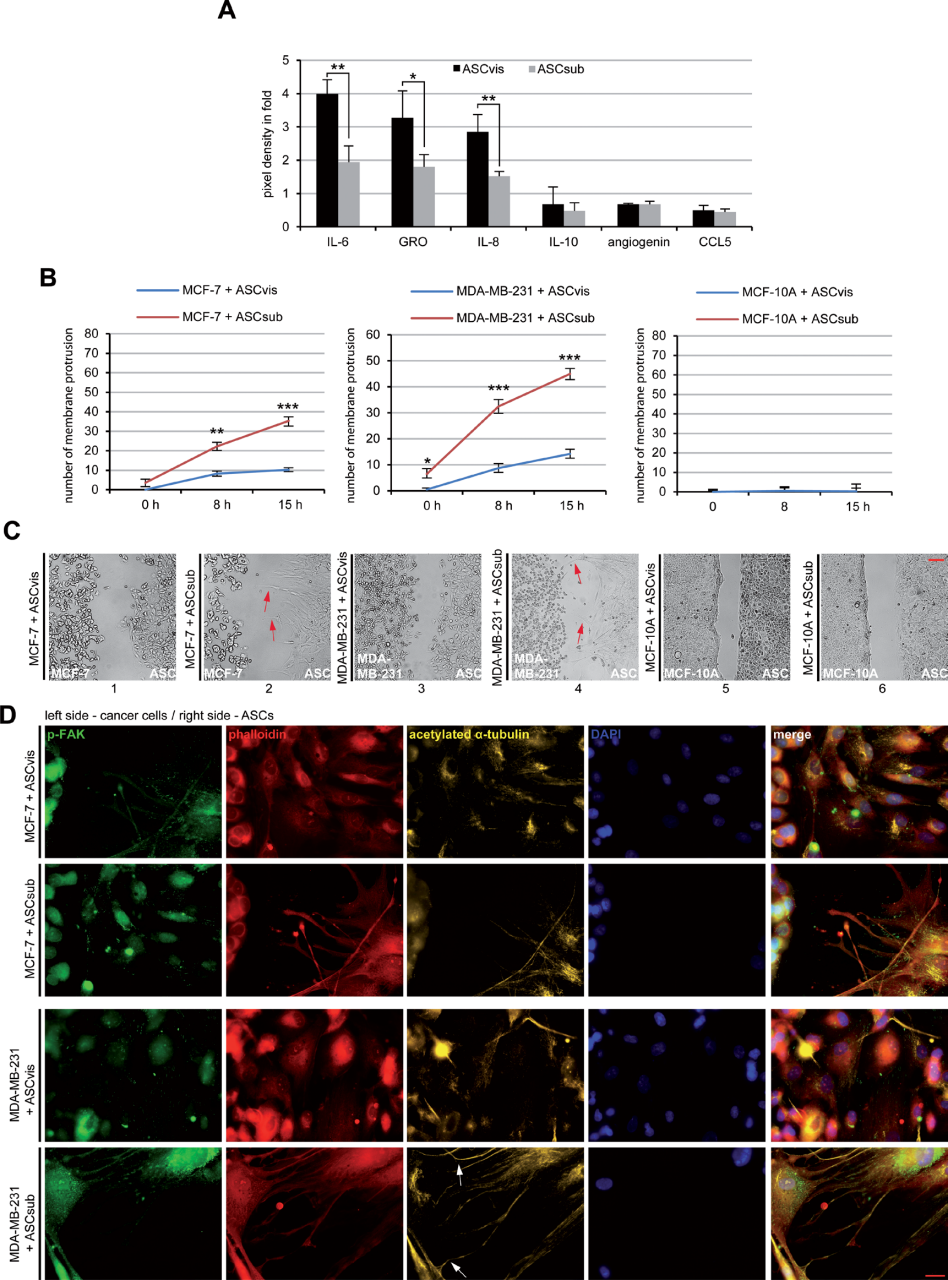
ASCs interact with cancer cells directly and indirectly **A.** The cytokine/chemokine array assay. The factors were measured in the supernatants of visceral ASCs (ASCvis) and subcutaneous ASCs (ASCsub) cultured for 3 days by using a human cytokine antibody array. The six most prominent chemokines/chemokines are demonstrated. The results, relative to the positive control provided by the array, are based on three independent experiments with ASCs obtained from three different donors and presented as mean ± SEM. **B.** Evaluation of membrane protrusion of ASCs toward MCF-7, MDA-MB-231 and MCF-10A cells. The results are based on three independent experiments with ASCs from three different donors and shown as mean ± SEM. **C.** Representatives of ASCs homing to breast cancer cell lines. Red arrows indicate membrane protrusions of ASCs toward breast cancer cells. Normal mammary epithelial MCF-10A cells served as negative control. Scale bar: 250 μm. **D.** Immunofluorescence staining of the migration front between ASCs and MCF-7 or MDA-MB-231 cells. Both cell types were stained for p-FAK, phalloidin, acetylated α-tubulin and DNA. White arrows depict the connections stabilized by acetylated α-tubulin between ASCs and breast cancer cells. Scale bar: 25 μm.

As mesenchymal stem cells are a source of many secreted cytokines, chemokines and growth factors [13], we analyzed next these factors in conditioned media from cultured ASCs with human cytokine arrays. Indeed, the array assay underscored the secretion of various cytokines, chemokines and growth factors by both types of ASCs (Table 3), especially IL-6, GRO, IL-8, IL-10, agiogenin and CCL5 showed high expression levels (Figure 2A). The secretion pattern of visceral and subcutaneous ASCs was very similar, however, the expression level varied between these two types of ASCs. Compared to subcutaneous ASCs, visceral ASCs were clearly more capable of secreting these factors (Table 3 and Figure 2A). Most of the detected secreted factors are known to be involved in cancer cell proliferation and progression [14].

**Table 3:** Cytokines/chemokines/adipokines in the supernatant of ASCs

ASC-secreted factors	visceral	subcutaneous	ASC-secreted factors	visceral	subcutaneous
CCL2	0.91 ± 1.02	0.56 ± 0.52	IL-2	0.02 ± 0.03	0.03 ± 0.05
TNF-α	0.19 ± 0.15	0.38 ± 0.38	CCL8	0.03 ± 0.04	0.16 ± 0.23
IL-3	0.20 ± 0.08	0.29 ± 0.17	IL-4	0.01 ± 0.02	0.02 ± 0.03
TNF-β	0.13 ± 0.04	0.24 ± 0.19	CCL7	0.01 ± 0.02	0.04 ± 0.05
CXCL5	0.48 ± 0.37	0.08 ± 0.12	EGF	0.09 ± 0.04	0.17 ± 0.14
IL-6	3.99 ± 0.43	1.94 ± 0.49	IL-5	0.01 ± 0.02	0.16 ± 0.11
angiogenin	0.68 ± 0.03	0.68 ± 0.09	IGF-I	0.03 ± 0.05	0.02 ± 0.03
IL-7	0.33 ± 0.31	0.22 ± 0.10	CCL22	0.06 ± 0.08	0.07 ± 0.09
oncostatin M	0.31 ± 0.06	0.35 ± 0.10	CSF1	0.22 ± 0.13	0.04 ± 0.05
IL-8	2.85 ± 0.52	1.52 ± 0.14	CXCL9	0.02 ± 0.03	0.02 ± 0.03
GRO	3.27 ± 0.81	1.80 ± 0.37	GM-CSF	0.03 ± 0.05	0.06 ± 0.08
IL-10	0.68 ± 0.52	0.48 ± 0.24	CCL15	0.03 ± 0.05	0.20 ± 0.14
CCL5	0.49 ± 0.16	0.45 ± 0.09	thrombopoietin	0.02 ± 0.02	0.03 ± 0.04
GRO-α	0.38 ± 0.35	0.06 ± 0.08	VEGF	0.02 ± 0.03	0.03 ± 0.05
SDF-1	0.13 ± 0.07	0.12 ± 0.04	IL-12	0.03 ± 0.04	0.03 ± 0.05
IL-1α	0.12 ± 0.06	0.59 ± 0.69	SCF	0.03 ± 0.04	0.04 ± 0.06
CCL17	0.14 ± 0.04	0.26 ± 0.21	PDGFB	0.02 ± 0.03	0.02 ± 0.03
IL-1β	0.13 ± 0.08	0.56 ± 0.68	CCL1	0.01 ± 0.02	0.02 ± 0.03
INF-γ	0.12 ± 0.12	0.30 ± 0.34	IL-13	0.01 ± 0.02	0.02 ± 0.03
TGF-β1	0.12 ± 0.08	0.21 ± 0.19	leptin	0.04 ± 0.05	0.03 ± 0.05
IL-15	0.02 ± 0.02	0.02 ± 0.03			

Relative chemokine secretion ratio of ASCs. The signal of the positive control was assigned as 1.

Abbreviations: CCL, C-C motif chemokine; TNF, tumor necrosis factor; IL, interleukin; CXCL, C-X-C motif chemokine; GRO, growth-regulated protein; SDF, stromal cell-derived factor 1; TGF, transforming growth factor; EGF, epidermal growth factor; IGF, insulin-like growth factor; CSF, colony-stimulating factor; GM-CSF, granulocyte-macrophage colony-stimulating factor; VEGF, vascular endothelial growth factor; SCF, stem cell factor; PDGF, platelet-derived growth factor.

Many studies have reported a tropism of ASCs toward cancer cells [15]. To underline this observation, we performed attraction assays, where we seeded visceral/subcutaneous ASCs and breast cancer cells in separated chambers of a culture insert with a defined cell free gap between these two chambers. The number of membrane protrusions of ASCs toward cancer cells, including lamellipodia and filopodia, was evaluated evidencing the tropism of ASCs. Both types of ASCs, especially subcutaneous ASCs, showed an increased homing ability toward breast cancer cells MCF-7, MDA-MB-231, fibroblasts and cervical carcinoma HeLa cells during the time period of 15 h (Figure 2B and 2C, and Figure S2A and B). By contrast, the normal mammary epithelial MCF-10A cells were not able to attract these two types of ASCs (Figure 2B, right panel, and Figure 2C, panel 5 and 6). To corroborate these results, we stained MCF-7 and MDA-MB-231 cells after 15 h for migration markers acetylated α-tubulin, phospho-Focal Adhesion Kinase (p-FAK) and phalloidin. The staining of phalloidin as well as p-FAK illustrated cytoskeleton protrusion of both ASC types, in particular subcutaneous ASCs, toward MCF-7 or MDA-MB-231 cells (Figure 2D). Interesting, the staining of acetylated α-tubulin was highly intensified on the connections between ASCs and breast cancer cells, likely to stabilize these new formed interactions (Figure 2D). Collectively, the data indicate that ASCs can interact with cancer cells directly *via* cell-cell connections and indirectly by the secretion of various soluble factors.

### Enhanced proliferation of breast cancer cells following direct co-culture with ASCs

**Figure 3:**
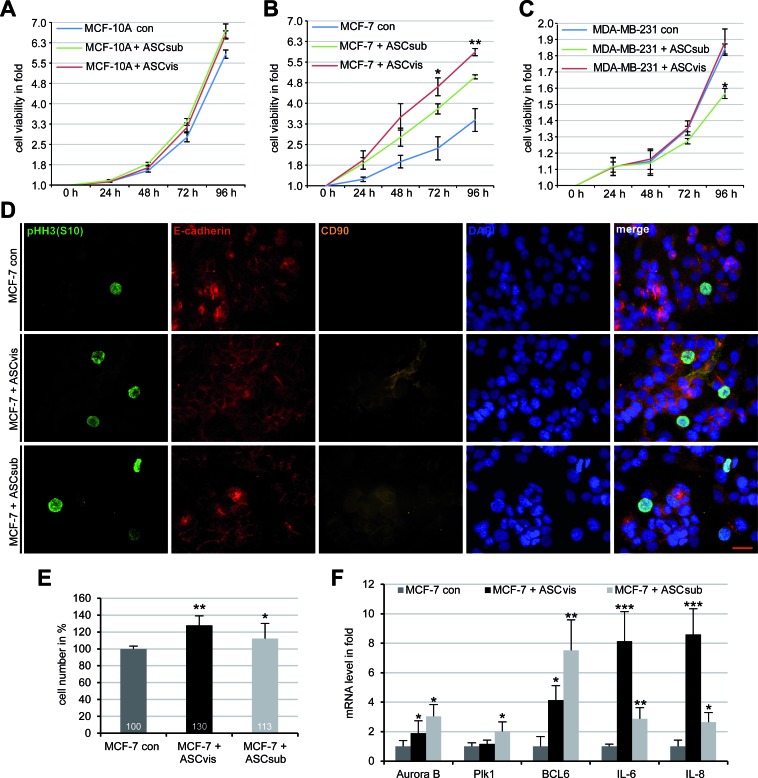
MCF-7 cells proliferate strongly upon direct co-culturing with ASCs, associated with increased mRNA expression of BCL6, IL-6 and IL-8 **A.-C.** Cell viability assay. MCF-10A **A.**, MCF-7 **B.** and MDA-MB-231 **C.**, in the presence or absence of 20% subcutaneous ASCs (ASCsub) or visceral ASCs (ASCvis), were seeded in 96-well plates. Cell viability was measured *via* CellTiter-Blue^®^ assay. The results are based on three independent experiments with ASCs from three different donors and presented as mean ± SEM. *p < 0.05, **p < 0.01. **D.** MCF-7 cells incubated with ASCs for 96 h were stained for phospho-histone H3 (pHH3, S10), a mitotic marker, E-cadherin, CD90 as ASC marker and DNA. Non-treated MCF-7 cells were taken as control. Representatives are shown. Scale bar: 25 μm. **E.** Cell number of Ruby-H2B MCF-7 cells co-cultured with 20% visceral ASCs or subcutaneous ASCs, evaluated by flow cytometry. The results are based on three independent experiments with ASCs from three different donors and shown as from three different donors and shown as mean ± SEM (*n* = 3). *p < 0.05, **p < 0.01. **F.** The gene expression of Ruby-H2B MCF-7 cells cultured alone or co-cultured with ASCs. The results are presented as mean ± SD and the values represent absolute mRNA levels relative to the standard curve. *p < 0.05, **p < 0.01, ***p < 0.001.

To address if ASCs have an effect on the proliferation of cancer cell lines, we performed cell viability assays. To determine the optimal ASC concentration, cell viability assays were performed with MCF-7 cells in the presence of increased ASC concentrations (5%, 10%, 15%, 20%, 30%). 20% of ASCs was taken for further investigations as it displayed the strongest promotion in MCF-7 cell proliferation (Figure S2C). In a direct co-culture manner with 20% of ASCs, the viability of breast cancer cells together with ASCs was measured at indicated time points. Compared to normal mammary epithelial cells MCF-10A (Figure 3A), low malignant breast cancer cells MCF-7 expanded significantly faster in the presence of either visceral or subcutaneous ASCs (Figure 3B). By contrast, visceral ASCs affected hardly and subcutaneous ASCs even inhibited proliferation of the highly metastatic breast cancer cells MDA-MB-231 (Figure 3C). The stimulatory effect of ASCs on MCF-7 was further underscored by an increase in the mitotic marker phosphorylated histone H3 (pHH3, S10) after five days of direct co-culture with visceral or subcutaneous ASCs (Figure 3D). To further underscore these data, Ruby-H2B labelled MCF-7 cells and EGFP-H2B marked MDA-MB-231 cells were directly co-cultured with ASCs for up to five days and the viability of the fluorescent cells were evaluated by flow cytometry. Compared to control Ruby-H2B MCF-7 cells, the cell number of co-cultured Ruby-H2B MCF-7 with visceral ASCs and subcutaneous ASCs was increased by 30% and 13%, respectively (Figure 3E). Again, proliferation of EGFP-H2B MDA-MB-231 cells was even slightly reduced in the presence of visceral or subcutaneous ASCs (Figure S2D). These different outcomes between MCF-7 and MDA-MB-231 cells might be ascribed to their individual proliferation pattern associated with their receptor composition: MDA-MB-231 cells proliferate autonomously representing a highly invasive cell type with a triple negative composition for estrogen receptor (ER), progesterone receptor (PR) and human epidermal growth factor receptor 2 (HER2), whereas less malignant MCF-7 cells, resistant to apoptosis due to the lack of caspase-3, proliferate upon stimulation with expression for all ER, PR and HER2 [16].

To study the impact of the direct co-culturing on the mRNA expression, total RNA was isolated from co-cultured and sorted Ruby-H2B MCF-7 cells and 12 genes related to the cell cycle were analyzed. Five of them were increased compared to that in control Ruby-H2B MCF-7 cells (Figure 3F) whereas the remaining 7 genes p21, NANOG, p53, RAC1, SOX2, HIF1α, MMP9, Aurora A and cyclin B1 were hardly affected (data not shown). The mRNA of Aurora B and Polo-like kinase 1 (Plk1), two important mitotic genes, increased slightly in Ruby-H2B MCF-7 cells co-cultured with visceral ASCs (Figure 3F). More interestingly, the mRNA expression level of the transcription suppressor B-cell lymphoma 6 (BCL6) and cytokines IL-6 and IL-8 were highly increased (Figure 3F). All three are known to be involved in multiple cellular processes like cell cycle regulation, proliferation, tumor initiation and progression [17-19].

### ASCs induce the epithelial-to-mesenchymal transition by indirect co-culturing

**Figure 4:**
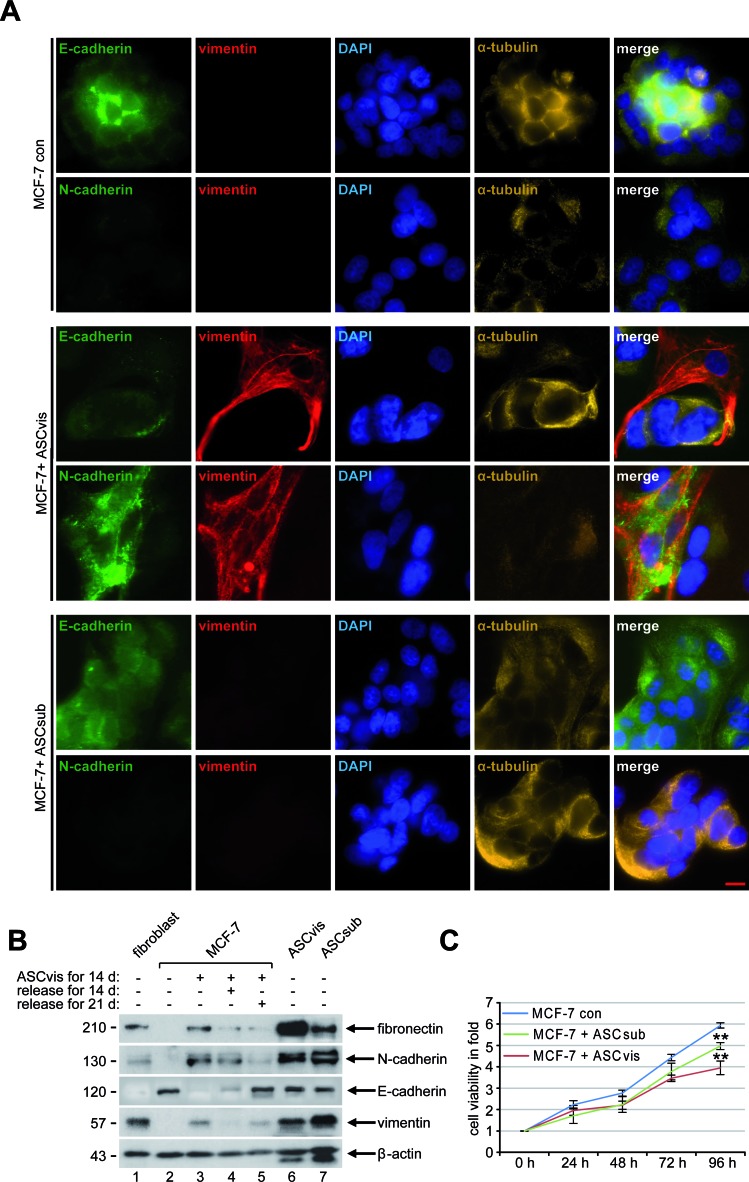
Indirect co-culturing with ASCs results in EMT in MCF-7 cells **A.** Immunofluorescence staining. MCF-7 cells were stained for the epithelial marker E-cadherin, the mesenchymal markers vimentin and N-cadherin, DNA and α-tubulin after 14 days of indirect co-culture with visceral ASCs. Representatives are depicted. Scale bar: 25 μm. **B.** Western blot analysis. Cellular lysates were prepared from MCF-7 cells, non-treated as control (con), indirectly co-cultivated with visceral ASCs (ASCvis) for 14 days, and co-cultured MCF-7 cells released for 14 and 21 days afterwards. Lysates from untreated fibroblasts, visceral ASCs or subcutaneous ASCs were taken as positive control cells. β-actin served as loading control. **C.** Cell viability assay. MCF-7 cells indirectly co-cultured with ASCs were seeded in 96-well plates and their viability was evaluated *via* a CellTiter-Blue^®^ assay. The results are based on three independent experiments with ASCs obtained from three different donors and presented as mean ± SEM. MCF-7 cells without co-culture served as control (con).

To address if ASCs could also indirectly influence cancer cells, an indirect co-culture experiment was performed in a transwell system. Breast cancer cells and ASCs were seeded in individual chambers of a transwell system for up to 14 days, where they had no direct cell-cell contact but they were able to exchange their media and secreted factors. The morphology of MCF-7 cells was visualized at indicated time points and the first spindle-like cell shape was observed after 4 days of indirect co-culturing (Figure S3A). During 14 days of indirect co-culture most MCF-7 cells lost their characteristic epithelial morphology with apical-basal polarity and gained a mesenchymal phenotype with spindle-shaped morphology (Figure S3A, day 7 to day 14), a process referred to as the epithelial-to-mesenchymal transition (EMT). To test if EMT is reversible, mesenchymal-like MCF-7 cells were then cultured alone without ASCs and the cell morphology was monitored at indicated time points. Interestingly, a timely decrease in mesenchymal-like and an increase in epithelial-like MCF-7 cells were observed (Figure S3B). To underscore this observation, cells were stained for confocal microscopy. Indeed, the analysis revealed a membrane and cytoplasmic distributed expression of N-cadherin and vimentin in MCF-7 cells co-cultured with visceral ASCs for 14 days (Figure 4A, 3^rd^ and 4^th^ panel). E-cadherin was still detectable above the background levels in these cells, but disappeared from the plasma membrane with a diffuse distribution in the cytoplasm (Figure 4A, 3^rd^ and 4^th^ panel). A similar morphologic change and expression of vimentin and E-cadherin were also observed in MCF-10A cells after incubation with visceral ASCs (Figure S3C). In contrast, MCF-7 control cells as well as MCF-7 cells co-cultured with subcutaneous ASCs for 14 days still displayed a strong expression of E-cadherin at the membrane and in the cytoplasm, with nearly no detectable N-cadherin and vimentin (Figure 4A, 1^st^ and 2^nd^ panel and 5^th^ and 6^th^ panel). To further corroborate these results, cells were harvested at indicated time points for Western blot analysis. MCF-7 cells indirectly co-cultured with visceral ASCs were demonstrative for mesenchymal cell markers, with high expression levels of fibronectin, N-cadherin and vimentin (Figure 4B, 1^st^ and 2^nd^ row, and 4^th^ row, lane 3), as observed in ASCs (Figure 4B, 1^st^ and 2^nd^ row, and 4^th^ row, lane 6 and 7), and with losing E-cadherin (Figure 4B, 3^rd^ row, lane 3). Also the reversibility of this process could be observed in mesenchymal-like MCF-7 cells cultured alone for 14 and 21 days, with a decrease in fibronectin and N-cadherin and an increase in E-cadherin (Figure 4B, 1^st^ to 3^rd^ row, lane 4 and 5), comparable to MCF-7 control cells (Figure 4B, 1^st^ to 3^rd^ row, lane 2).

Nevertheless, subcutaneous ASCs were able to induce EMT in MCF-7 after 21 days of indirect co-culture, displayed by a changed morphology, increased vimentin and decreased E-cadherin expression (Figure S3D). The observation, subcutaneous ASCs induced EMT with a significantly decreased rate and a longer time period in MCF-7 cells (Figure S3E), might be explained by reduced secretion of the most detected cytokines from subcutaneous ASCs compared to visceral ASCs (Figure 2A).

It is well known that EMT reduces very often cell proliferation [20-22]. To functionally corroborate the EMT phenotype induced by ASCs, we performed cell viability assays. MCF-7 control cells and MCF-7 cells indirectly co-cultured with ASCs for 14 days were seeded into a 96-well plate for up to 96 h. While MCF-7 cells indirectly co-cultured with visceral ASCs showed a reduced proliferation rate of 33.5%, MCF-7 indirectly incubated with subcutaneous ASCs displayed a reduction of 16.2% at 96 h compared to MCF-7 control cells (Figure 4C). Collectively, the data imply that the indirect co-culture with ASCs induces EMT in MCF-7 cells and this transition is reversible associated with significant changes in their cytoskeleton composition, morphological changes and a reduced cell proliferation.

### The induced EMT is triggered by multiple signaling cascades in MCF-7 cells

**Figure 5:**
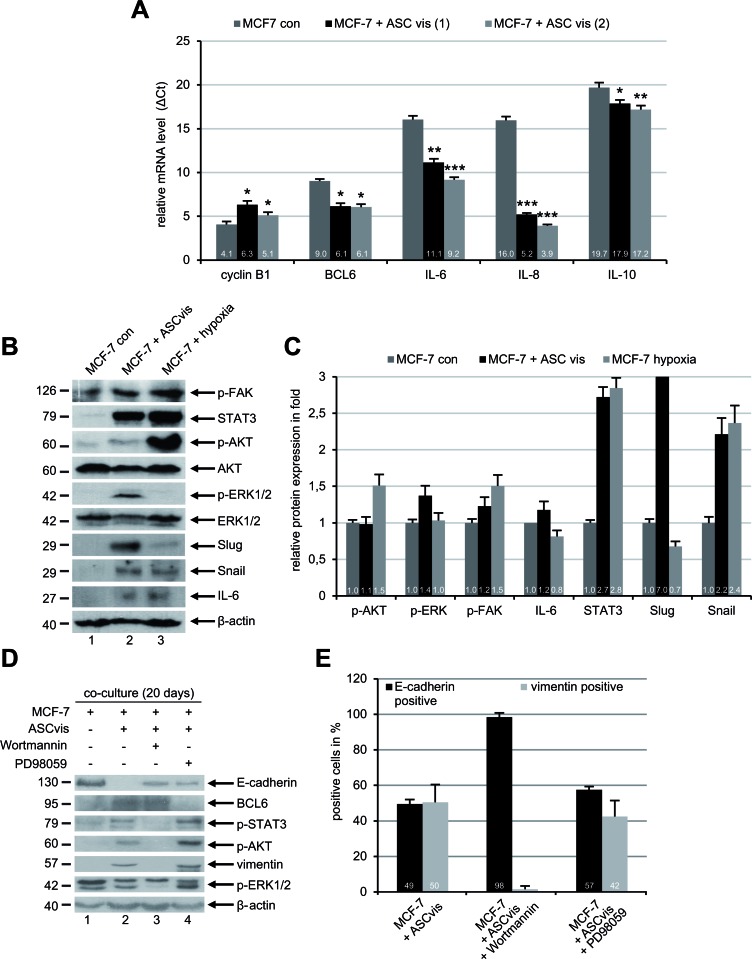
ASCs induce EMT in MCF-7 cells through multiple signaling pathways **A.** The mRNA expression levels of the mitotic marker cyclin B1, transcription suppressor BCL6 and cytokines IL-6, IL-8 and IL-10 were determined by real-time PCR in MCF-7 cells cultured alone or in co-culture with visceral ASCs (ASCvis) from two donors. The results are presented as mean ± SD. *p < 0.05, **p < 0.01, ***p < 0.001. Note: each gene expression is demonstrated by ΔCt value, which is normalized to endogenous GAPDH and is reversely related to the amount of target mRNA. **B.** Western blot analyses with indicated antibodies. Cellular lysates were prepared from MCF-7 control cells and MCF-7 cells indirectly co-cultured with visceral ASCs for 14 days. Lysates from MCF-7 cells exposed to hypoxia (2% O_2_) were taken as positive mesenchymal control. β-actin served as loading control. **C.** Quantification of Western blot analyses in **B.**, relative to corresponding β-actin signal. The results are based on two independent experiments and presented as mean ± SEM. The value in MCF-7 control cells is defined as 1 fold. The quantification was performed with ImageJ. **D.** MCF-7 cells were cultured alone or with visceral ASCs in the presence of a PI3K inhibitor Wortmannin (50 nM) or a MAPK inhibitor PD98059 (10 μM). After 20 days cellular lysates were prepared for Western blot analyses with indicated antibodies. β-actin served as loading control. **E.** The treated cells in **D.** were fixed and stained for the epithelial marker E-cadherin and mesenchymal marker vimentin. Positive cells were counted (*n* = 200 cells for each condition) and the results are presented as mean ± SD (*n* = 3).

Numerous cytokines and chemokines have been reported to be involved in the EMT process of cancer cells *via* multiple signaling pathways [23]. To address this issue, we measured at first the mRNA levels of several cytokine genes in MCF-7 cells after co-culture with visceral ASCs. The gene expression is demonstrated by ΔCt value, which is reversely related to the amount of target mRNA. Interestingly, co-cultured MCF-7 cells showed a remarkable upregulation of IL-6, IL-8 and IL-10 mRNA compared to MCF-7 control cells (Figure 5A). The cyclin B1 mRNA level was slightly reduced in the ASC co-cultured MCF-7 cells reflecting the reduced cell proliferation of EMT cells (Figure 5A). Of note, the gene expression of the transcription suppressor BCL6 was also increased (Figure 5A). To look closely at EMT, we performed further Western blot analysis with lysates from control MCF-7 cells, ASC co-cultured MCF-7 cells and MCF-7 cells exposed to hypoxia (2% O_2_), the late was taken as an EMT positive control. Interestingly, compared to control MCF-7 cells, visceral ASC co-cultured MCF-7 cells displayed an increase in transcription factors Snail, Slug and STAT3 associated with enhanced IL-6 (Figure 5B and 5C). Moreover, an activation of the phospho-AKT (p-AKT), p-ERK1/2 and p-FAK was also observed (Figure 5B and 5C).

To further elucidate which of these pathways is important for the induction of EMT, MCF-7 cells were co-cultured with visceral ASCs for 20 days in the presence of low concentrations of Wortmannin (50 nM), a potent phosphatidylinositol 3-kinase (PI3K) inhibitor [24], or PD98059 (25 μM) inhibiting the mitogen-activated protein kinase (MAPK) family [25]. Wortmannin blocked the EMT induction in MCF-7 co-cultured with visceral ASCs by displaying no increase in the expression of vimentin, p-AKT and p-STAT3 compared to MCF-7 control cells (Figure 5D, lane 1 and 3), indicating the activation of PI3K pathway is essential for the EMT process. On the other hand, the inhibition of the MAPK pathway with PD98059 hardly affected the EMT induction evidenced by increased vimentin, p-AKT and p-STAT3, and decreased E-cadherin (Figure 5D, lane 2 and 4). To corroborate these results, immunofluorescence staining was carried out. While 51% of visceral ASCs co-cultured with MCF-7 cells were positive for the mesenchymal marker vimentin, only few of these cells expressed vimentin in the presence of Wortmannin (Figure 5E). Again, the MAPK inhibitor PD98059 was not able to affect the EMT process in MCF-7 cells co-cultured with visceral ASCs showing increased vimentin expression (Figure 5E). These data provide evidence that EMT of MCF-7 cells induced by ASCs is mediated by multiple pathways, yet predominantly by the PI3K pathway known to be activated by IL-6 and IL-8 [23], which are highly secreted by ASCs (Figure 2A).

### Indirect co-culture with ASCs promotes malignancy of breast cancer cells

**Figure 6:**
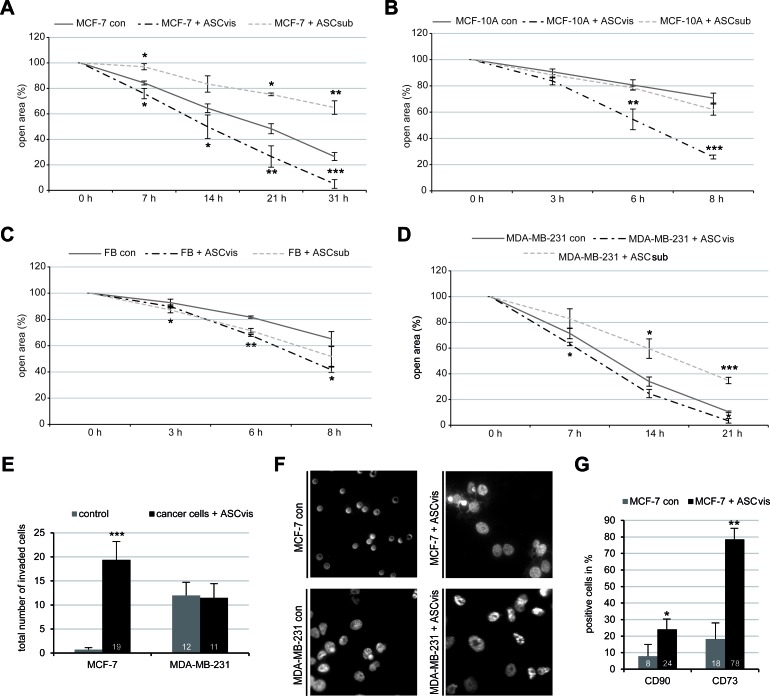
ASCs increase malignant properties of breast cancer cells Wound healing/migration assay was performed with MCF-7, MCF-10A, MDA-MB-231 and fibroblasts in the presence or absence of visceral ASCs (ASCvis) or subcutaneous ASCs (ASCsub) and pictures were taken at indicated time points to document the migration front. **A.-D.** Quantification of the open area between both migration fronts at various time points in MCF-7 **A.**, MCF-10A **B.**, fibroblast (FB) **C.** and MDA-MB-231 cells **D.**, using the AxioVision SE64 Rel. 4.9 software (*n* = 5 visual fields of 1350 × 1800 μm^2^ for each condition). The cell-free area of each individual condition at 0 h was assigned as 100%. The results are based on three independent experiments with ASCs from three different donors and presented as mean ± SEM. *p < 0.05, **p < 0.01, ***p < 0.001. **E.** Invasion assay *via* a transwell system. Quantification of invaded MCF-7 and MDA-MB-231 cells cultured alone or co-cultured with visceral ASCs. The number of invaded breast cancer cells cultured alone was assigned as 1 fold. The results are presented as mean ± SEM (*n* = 5 visual fields of 170 × 225 μm^2^ for each condition). ***p < 0.001. **F.** Representatives of invaded MCF-7 and MDA-MB-231 cells cultured alone or indirectly cultured with visceral ASCs for 14 days. Scale bar: 20 μm. **G.** MCF-7 cells cultured alone or co-cultured with visceral ASCs for 14 days were fixed and stained for mesenchymal stem cell surface markers CD73 and CD90. Positive cells were evaluated by flow cytometry. The results are obtained from three independent experiments with ASCs from three different donors and presented as mean ± SEM. *p < 0.05; **p < 0.01.

We were then interested in the invasive property of breast cancer cells co-cultured with ASCs. A wound-healing/migration assay was performed with different cell lines indirectly co-cultured with ASCs. After co-culturing with visceral ASCs, breast cancer cells MCF-7 and MDA-MB-231, together with normal mammary epithelia cells MCF-10A and fibroblasts, displayed a significantly increased wound healing capacity compared to control cells (Figure 6A-6D and Figure S4A-S4D). In the context of co-culturing with subcutaneous ASCs, intriguingly, non-tumorigenic cell lines like MCF-10A and fibroblasts showed a slightly increased wound healing capacity, whereas tumorigenic MCF-7 cells and metastatic MDA-MB-231 cells exhibited a slower migration rate compared to control cells (Figure 6A-6D and Figure S2A-S2D). To examine the invasive feature of breast cancer cells, ASC co-cultured cells were used for a well-established invasion assay [26]. As expected, MCF-7 control cells were not able to invade the matrigel layer of the invasion chamber, whereas MCF-7 co-cultured with visceral ASCs displayed a highly significant increase in the number of invading cells (Figure 6E and 6F). Intriguingly, visceral ASCs stimulated hardly the invasive capability of MDA-MB-231 cells, which are already highly invasive metastatic breast cancer cells (Figure 6E and 6F). Thus, the data imply that ASCs promote cell migration of different cell lines and facilitates the invasion mainly in non/less invasive breast cancer lines such as MCF-7.

The recently emerged concept of cancer stem cells as a cell state not as a cell type [27] led to the question, if the indirect co-culture with ASCs is sufficient to induce the expression of mesenchymal stem cell markers in MCF-7 cells. To answer this question, visceral ASC co-cultured MCF-7 cells were measured by flow cytometry for mesenchymal stem cell markers CD90 and CD73, which have been shown to be correlated with tumor growth, metastasis and drug resistance in breast cancer cells [28, 29]. Indeed, the number of CD90 and CD73 positive stained MCF-7 cells was increased by 16% and 60%, respectively, after co-culture with visceral ASCs, compared to MCF-7 control cells (Figure 6G). These results further support the notion that ASCs could change relatively differentiated MCF-7 into more progenitor state conferring the malignant properties.

### ASC signaling confers resistance to Plk1 inhibitors in MCF-7 cells

**Figure 7:**
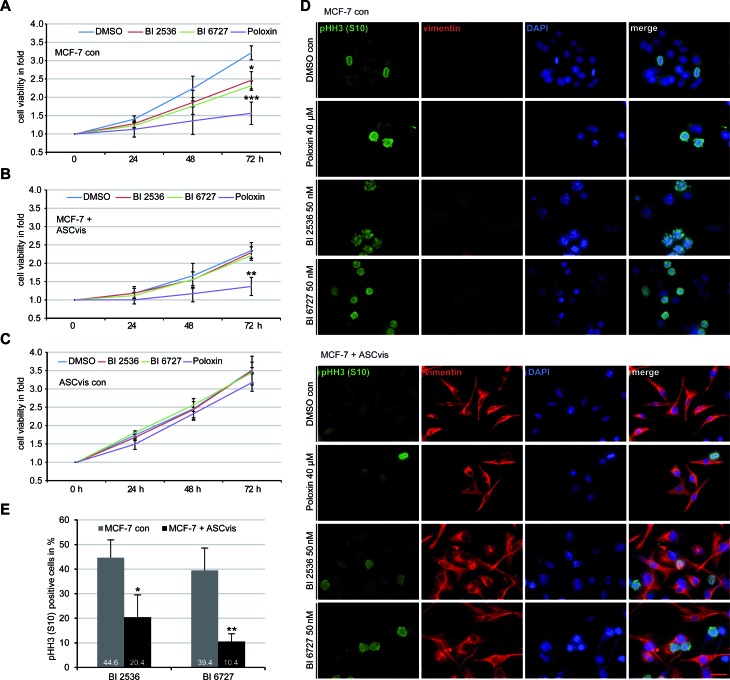
EMT-like MCF-7 cells are resistant to BI compounds **A.**-**C.** Cell viability assay. MCF-7 cells **A.**, MCF-7 cells incubated with visceral ASCs for 14 days **B.** and visceral ASCs alone **C.** were seeded in 96-well plates and treated with Plk1 inhibitors BI 2536 (50 nM), BI 6727 (50 nM) or Poloxin (40 μM) and their viability was measured at indicated time points *via* CellTiter-Blue^®^ assay. DMSO treated cells served as vehicle control. The results are obtained from three independent experiments with ASCs obtained from three different donors and presented as mean ± SEM (*n* = 3). *p < 0.05, **p < 0.01, ***p < 0.001. **D.** The treated cells were stained for pHH3 (S10), vimentin and DNA. The representatives are shown (upper panel: MCF-7 control cells; lower panel: MCF-7 cells co-cultured with visceral ASCs). Scale bar: 25 μm. **E.** Quantification of positive pHH3 (S10) staining in MCF-7 control cells or in mesenchymal-like MCF-7 cells treated with BI compounds for 48 h. The results are from three independent experiments with ASCs from three different donors and presented as mean ± SD (*n* = 3). *p < 0.05, **p < 0.01.

EMT is linked to drug resistance and poor prognosis for patients [30]. Plk1 is one of the most promising targets for molecular cancer treatment and its inhibitors block efficiently proliferation of various cancer cells including MDA-MB-231 and MCF-7 cells [31, 32]. We wondered if the EMT induction renders MCF-7 resistant to Plk1 inhibitors. MCF-7 cells, mesenchymal-like MCF-7 cells induced by co-culturing with visceral ASCs, and visceral ASCs were subjected to Plk1 inhibitors BI 2536, BI 6727 and Poloxin, and the cell viability and the mitotic index were examined. Compared to control MCF-7 cells (Figure 7A), mesenchymal-like MCF-7 cells were obviously resistant to BI 2536 and BI 6727 by showing no significant proliferative inhibition (Figure 7B). Interestingly, Poloxin, an inhibitor targeting the polo-box binding domain of Plk1, displayed an inhibitory effect on those cells (Figure 7B), which requires further investigation. As expected, visceral ASCs responded hardly to Plk1 inhibitors (Figure 7C). Treated cells were further stained for mesenchymal marker vimentin and the mitotic marker pHH3 (S10) for microscopy. Compared to control MCF-7 cells (Figure 7D, upper panel), the pHH3 (S10) positive cells were clearly reduced in ASC co-cultured MCF-7 cells (Figure 7D, lower panel), which were vimentin positive (Figure 7D, lower panel) indicating their mesenchymal-like phenotype. Further evaluation corroborated a strong reduction of the pHH3(S10) staining in mesenchymal-like MCF-7 cells upon BI compound treatment (Figure 7E). These results point to the notion that MCF-7 cells co-cultured with ASCs acquire resistance to Plk1 inhibitors BI 2536 and BI 6727, likely obtained by the changed cytoskeleton composition and decreased proliferation rate [23].

## DISCUSSION

The tumor microenvironment plays a crucial role in cancer development and progression [33]. To ensure the safety usage of ASCs in new regenerative therapy, it is fundamental to understand the molecular interactions between ASCs and cancer cells. A great body of work has been done, which has shed light on this issue and also raised the safety concerns [8-10, 34-38]. In the current study, we have isolated ASCs from subcutaneous and visceral adipose tissues of the same donor and systematically characterized the features of these paired ASCs and addressed their impact on breast cancer cells.

We show that both subcutaneous and visceral ASCs express the cell surface markers characteristic of the mesenchymal stem cells. They display however distinct cell morphology in culture, at least in the early passages: ASCs from subcutaneous adipose tissues incline to be fibroblastic-like, whereas their counterparts from visceral adipose tissues tend to be “epithelial”-like. Moreover, visceral ASCs are more competent of secreting soluble factors, differentiating into other cell types and impact more strongly on other cells, whereas subcutaneous ASCs are more capable of homing toward breast cancer cells. The differences between subcutaneous ASCs and visceral ASCs could be ascribed to their different original sources [39] and varied surroundings. In particular, the surrounded adipocytes, namely the adipocytes in subcutaneous and in visceral adipose tissue, exert definitely distinct physiological functions [40]. The data might imply diverse roles of these ASCs *in vivo*: subcutaneous ASCs could be more mobile functioning in far organs and places, whereas visceral ASCs could work more locally in near areas. Despite differing in time and in efficiency, nevertheless, both ASCs differentiate into other cells types, home toward cancer cells and establish direct cell-cell contacts. Additionally, both ASCs secret various factors involved in autocrine and paracrine regulation and linked to tumor initiation, growth and metastasis [41].

Furthermore, we show that, in a direct co-culture manner, visceral ASCs promote proliferation of the low malignant breast cancer line MCF-7, but hardly affect the cell viability of the invasive breast cancer cell line MDA-MB-231. Compared to visceral ASCs, subcutaneous ASCs are much less competent to stimulate proliferation of MCF-7 cells and even weakly inhibit expansion of MDA-MB-231 cells. Interestingly, both ASCs impact also slightly proliferation of normal mammary epithelial cells MCF-10A. The reduced effect of subcutaneous ASCs might be explained by a reduced expression of various cytokines like IL-6 and IL-8 known to be involved in breast cancer proliferation [42, 43]. The immovableness of MDA-MB-231 cells could be due to their receptor composition, the negative expression of ER, PR and HER2 [16], in line with the notion that triple negative breast cancer cells respond differently to Notch, Hedgehog, Wnt/β-catenin and TGFβ signaling pathways [44], which are activated by ASCs [45, 46]. Furthermore, direct cell-cell incubation induces an upregulation of mitotic genes Aurora B and Plk1, cytokine genes IL-6 and IL-8 and the oncogene BCL6 known for their tumor promoting functions [17, 42, 47]. Intriguingly, indirect incubation *via* a transwell system seems to be inefficient to promote proliferation, indicating that a direct cell-cell contact, possibly combined with the extracellular matrix associated growth factors, is required for an effective proliferation.

Moreover, our results reveal that, despite varied capability, both types of ASCs are able to induce EMT in breast cancer cells by indirect co-culturing. This process is illustrated by a changed morphology, altered cytoskeleton composition and a reduced proliferation rate. Along with the modification of the cytoskeleton, the mRNA levels of BCL6, IL-6, IL-8 and IL-10 are highly increased, which are all well documented to induce EMT by multiple pathways in diverse cell lines [47-50]. Of note, we show that BCL6, a crucial player involved in B cell-lymphoma [51], is increased in MCF-7 cells upon direct co-incubation with ASCs promoting proliferation, as described above, and upon indirect co-culture in a transwell system with ASCs facilitating EMT in MCF-7 cells. In line with previous observation [52], our data highlight that BCL6 is pivotal in progression of breast cancer cells stimulated by ASCs. Furthermore, Western blot analyses with lysates from mesenchymal-like MCF-7 cells display increased activation of the PI3K/AKT, ERK1/2 and p-FAK pathways, together with enhanced expression of the transcription factors Snail, Slug and STAT3, also known as key players in the EMT process [53]. The results from treatment with kinase inhibitors suggest that the PI3K/AKT pathway is required for the EMT induced by ASCs, whereas the MAPK pathway seems to be dispensable for this process unlike reported for human lens epithelial cells or ovarian cancer cells [54, 55]. Consequently, the EMT-like MCF-7 cells show a reinforced migration and invasion potential, and confer resistance to Plk1 inhibitors BI 2536 and BI 6727, in accordance with previous reports showing resistance to other chemotherapeutic drugs [56]. Along with EMT, MCF-7 cells express high levels of CD73 and CD90, two cell surface markers of mesenchymal stem cells, supporting the correlation between EMT and stemness [57]. Our results also suggest that low malignant breast cancer lines such as MCF-7 will be strongly impacted by ASCs, which enforce them into a more aggressive phenotype. In addition, subcutaneous ASCs, isolated from donors undergoing cesarean section, appear to be less competent in this study regarding their ability to promote proliferation and induce EMT in breast cancer cells. It has been reported that the reproductive state of donors hardly impacted the features of ASCs [58], it is still conceivable that various alterations in the pregnancy, like hormones, growth factors and metabolism, could affect ASCs and subsequently change their abilities. Further investigations are required to address this issue.

In summary, we demonstrate pro-tumorigenic effects of paired visceral and subcutaneous ASCs on proliferation, migration and invasion behavior of various cancer-derived cell lines. The interactions of ASCs with breast cancer cells, *via* direct cell-cell communication or indirect connection by secreting various cytokines, chemokines and growth factors, increase the expression of diverse cytokines, transcription factors and cell-surface proteins, induce EMT in breast cancer cells *via* activating multiple signaling pathways and render them resistant to Plk1 inhibition. More preclinical investigations are warranted to clarify these pro-tumorigenic effects of ASCs in more molecular depth. In parallel, more clinical studies with prolonged follow-up are required to address cancer recurrence in patients treated with ASC-enriched fat grafts.

## MATERIALS AND METHODS

### Human adipose-derived stem cell (ASC) isolation, cell culture, differentiation and inhibitors

Ethics approval was obtained from the Ethics Committee of the university Hospital Frankfurt and informed written consent was obtained from all donors. Visceral (omental) and subcutaneous (abdominal) adipose tissues were taken from women undergoing cesarean section. ASCs were isolated as described [11] with modifications. In brief, obtained visceral and subcutaneous adipose tissues were immediately washed, minced to small pieces, and digested with 1 mg/ml collagenase type I for 1 h at 37°C. Cells were pelleted by centrifugation at 700 g for 10 min and the remaining blood cells were lysed by addition of red blood cell lysis buffer (155 mM NH_4_Cl, 10 mM KHCO_3_, and 0.1 mM EDTA) for 10 min at 37°C. To remove the lysis buffer cells were centrifuged at 700 g for 10 min and filtered through a 100-m mesh filter to remove undigested adipose tissue. The remaining cells were centrifuged, seeded onto 6-cm cell cultures plates in DMEM containing 20% fetal bovine serum (FBS), 100 g/ml streptomycin, 100 U/ml penicillin, 2 mM l-glutamine, and 1 g/ml amphotericin-B and cultured under standard cell culture conditions. After 24 h, non-adherent cells were removed and the remaining cells were washed, cultured and expanded. Early passages (P2 to P5) of isolated ASCs were used for experiments.

To induce adipogenic differentiation, cells were cultured with StemMACS^TM^ AdipoDiff Media (Miltenyi Biotec, Gladbach) for 14 days. Cells were then fixed and stained for oil red O and adiponectin characteristic of adipocytes. For osteogenic differentiation, ASCs were incubated in StemMACS^TM^ OsteoDiff Media (Miltenyi Biotec) for 21 days, fixed and stained with 2% Alizarin Red S (pH 4.2) to visualize calcific deposition by cells of an osteogenic lineage. For neurogenic differentiation, cells were cultured in DMEM supplemented with 5 mM KCl, 2 μM valproic acid, 10 μM forskolin, 1 μM hydrocortisone, 5 μg/ml insulin, 0.5% ethanol and 200 μM butylated hydroxyanisole for 21 days, and cells were stained for class III beta-tubulin and doublecortin.

MCF-7, MDA-MB-231 and MCF-10A were obtained from ATCC and skin fibroblasts were a kind gift from our Department of Dermatology, Frankfurt. All cells were cultured as instructed. BI 2536 and BI 6727 were purchased from Selleck Chemicals LLC (Houston) and Poloxin was kindly provided by Dr. Berg (Leipzig University). DMSO was from Sigma-Aldrich (Taufkirchen), PD98059 from Merck Millipore (Darmstadt) and Wortmannin from Cell Signaling (Beverly).

### Cellular extract preparation, western blot analysis and cytokine array

Cellular lysates were prepared using RIPA buffer (50 mM Tris pH 8.0, 150 mM NaCl, 1% NP-40, 0.5% Na-desoxycholate, 0.1% SDS, 1 mM NaF, 1 mM DTT, phosphatase and protease inhibitor cocktail tablets (Roche, Mannheim)). Western blot analysis was performed as previously described [59, 60]. Following antibodies were used: Rabbit polyclonal antibodies against AKT, phospho-AKT (S473), phospho-FAK (Y397), STAT3 and phospho-STAT3 (Y705) (Cell Signaling), rabbit monoclonal antibodies against E-cadherin, N-cadherin, Slug and Snail (Cell Signaling), mouse monoclonal antibody against β-actin (Sigma-Aldrich), mouse monoclonal antibodies against BCL6 and vimentin (Dako, Hamburg), rabbit polyclonal antibody against ERK1/2 and rabbit monoclonal antibody against phospho-ERK1/2 (T202/Y204) (Merck Millipore, Darmstadt), rabbit monoclonal antibody against fibronectin and rabbit polyclonal antibody against IL-6 (Abcam, Cambridge).

For cytokine measurement in supernatants, visceral and subcutaneous ASC in passage 1 were cultured for 3 days to a confluence of 90%. The levels of chemokines, cytokines and growth factors in the culture media were determined by a human cytokine antibody array according to the manufacturer's instructions (Abcam). The chemiluminescent membranes were developed using the ChemiDoc^TM^ MP System (Bio-Rad, Munich) and the signal intensity was assessed with ImageJ 1.49i software (National Institutes of Health, USA) by determining the pixel intensity of the detected spots.

### Indirect immunofluorescence

Cells were seeded on Nunc^TM^ Lab-Tek^TM^ SlideFlask chambers from Thermo Fisher Scientific (Schwerte). Immunofluorescence staining was performed as described [61-63]. Briefly, cells were fixed for 10 min with methanol at −20°C or with 4% paraformaldehyde containing 0.1% Triton^TM^ X-100 for 15 min at room temperature. The following primary antibodies were used for staining: monoclonal mouse antibody against FITC-conjugated α-tubulin and mouse monoclonal antibody against acetylated α-tubulin (Sigma-Aldrich), rabbit polyclonal antibodies against pericentrin, mouse monoclonal antibody against adiponectin and rabbit monoclonal antibodies against CD90, E-cadherin and N-cadherin (Abcam), mouse monoclonal antibody against DCX, rabbit polyclonal antibody against CD73 and chicken polyclonal antibody against Tuj1 (GeneTex), mouse monoclonal antibody against vimentin (Dako), mouse monoclonal antibody against pHH3 (S10) (Merck Millipore), rabbit polyclonal antibody against phospho-FAK (Y397) (Cell Signaling), rat monoclonal antibody against α-tubulin (Biomol, Hamburg) and mouse monoclonal antibodies against CD14 and CD31 (Biolegend, Fell). Filamentous actin was stained using phalloidin-TRITC (Sigma-Aldrich) and DNA was visualized by using DAPI (4′,6-diamidino-2-phenylindole-dihydrochloride, Roche, Mannheim). The immunofluorescence slides were examined using an AxioObserver.Z1 microscope (Zeiss, Göttingen) or by a confocal laser scanning microscope (CLSM, Leica CTR 6500, Heidelberg).

For flow cytometry, cells were harvested with 0.25 % trypsin, fixed for 15 min with ice-cold 2% PFA at 4°C. Cells were washed twice with FCB (PBS, 0.2% Tween-20, 2% FCS) and stained with the following antibodies from eBioscience (Frankfurt am Main): FITC-conjugated anti-human CD90, PerCP-Cy5.5-conjugated anti-human CD90, PE-conjugated anti-human CD14, FITC-conjugated anti-human CD34, PerCP-Cy5.5-conjugated anti-human CD146, APC-conjugated anti-human CD106 and APC-conjugated anti-human CD31.

### Cell proliferation and flow cytometry

Cell proliferation assays were performed by using Cell Titer-Blue^®^ Cell Viability Assay (Promega, Mannheim) as described [32]. The ratio 1:5 between ASCs and cancer cells was used for direct co-culture experiments. MCF-7 and MDA-MB-231 cells were stably transfected with Ruby-H2B or EGFP-H2B and selected with geneticin (Sigma-Aldrich). The stable cell lines were verified by flow cytometry. Both cell lines were co-cultured with ASCs (5:1 ratio) in a 6-cm culture dish. After five days, cells were analyzed using a FACSCalibur^TM^ (BD Bioscience, Heidelberg). The percentages of EGFP or Ruby positive cancer cells were determined with BD CellQuest^TM^ Pro software (BD Bioscience).

To evaluate the mitotic fraction, MCF-7 cells and MCF-7 cells indirectly co-cultivated with visceral ASCs for 14 days, were seeded into slide flask chambers. After 8 h the cells were treated with 50 nM BI 2536 or 50 nM BI 6727 for 48 h. Cells were stained for phospho-histone H3 (pHH3, S10). Five pictures of each slide flask were taken and the experiments were performed in triplicate. The percentage of positive pHH3 (S10) cells were evaluated using the AxioVision SE64 Re. 4.9 software (Zeiss).

### Migration, invasion and attraction assay

Cell migration assays were performed with culture-inserts from ibidi (Martinsried). Culture-inserts (cell free gap of 500 μm) were placed in a 6-cm culture dish and both wells of each insert were filled with cell suspension. MCF-7 (6.5 × 10^4^), MDA-MB-231 (5.5 × 10^4^), fibroblasts (6.5 × 10^4^) and MCF-10A (5 × 10^4^) cells were seeded in each well of the culture-inserts, which were surrounded by visceral or subcutaneous ASCs. Culture-inserts were gently removed after at least 8 h. The cells were acquired and imaged at indicated time points with bright-field images. Four pictures of each insert were taken and the experiments were performed in triplicate. The open area was measured using the AxioVision SE64 Re. 4.9 software (Zeiss).

For attraction assay, cells were placed in a 6-cm culture dish and one well of each insert was filled with cell suspensions either with visceral or subcutaneous ASCs (5.5 × 10^4^) or with the investigated cells (MCF-7, MDA-MB-231, fibroblasts or MCF-10A). After 8 h, the culture-inserts were removed and the images were obtained at indicated time points. Cellular movement toward other migration front was evaluated using the AxioVision SE64 Re. 4.9 software (Zeiss). The experiments were independently performed three times.

For invasion assay, MCF-7 and MDA-MB-231 cells were indirectly co-cultured with visceral ASCs in a transwell chamber for at least 14 days. Cells were then seeded in 24-well transwell matrigel chambers according to the manufacturer's instructions (Cell Biolabs Inc, San Diego) and as previously reported [26]. Briefly, cells (MDA-MB-231, 7.5 × 10^4^; MCF-7, 12 × 10^4^) were seeded into the upper chamber of the transwell in 500 μl serum-free medium and the lower chamber was filled with 750 μl serum-free medium. After 12 h the medium of both chambers was discarded and the invasion assay was started by adding medium containing 10% fetal bovine serum for the next 24 h. Cells were fixed with ethanol and stained with DAPI. Invaded cells were counted with a microscope. The experiments were independently performed three times.

### RNA extraction and real-time PCR

Total RNAs of ASCs and cancer cells were extracted with RNeasy kits with column DNase digestion according to the manufacturer's instructions (QIAGEN, Hilden). Reverse transcription was performed using High-Capacity cDNA Reverse Transcription Kit (Applied Biosystems, Darmstadt) as instructed. The probes for Aurora A, Aurora B, BCL6, cyclin B1, IL-6, IL-8, IL-10, p21 and Plk1 were obtained from Applied Biosystems. Real-time PCR was performed with a StepOnePlus Real-time PCR System (Applied Biosystems). The data were analyzed using StepOne Software v.2.3 (Applied Biosystems) as described previously [60, 64] with two methods. First is the comparative Ct method: the relative quantity of target gene expression is determined by comparing a reference sample and an endogenous control to the target sample. This method displays the gene expression as ΔCt value, which is normalized to endogenous GAPDH and is reversely related to the amount of target mRNA. Second is the standard curve method which uses standards to determine the absolute quantity of target gene expression.

### Statistical analysis

Student's *t*-test (two tailed and paired or homoscedastic) was used to evaluate the significance of difference between different groups. Difference was considered as statistically significant when *p* < 0.05.

## SUPPLEMENTARY MATERIAL FIGURES



## Abbreviations

ASC: adipose-derived stem cell
MSC: mesenchymal stem cell
IL: interleukin
PI3K: phosphatidylinositol 3-kinase
BCL6: B-cell lymphoma 6
MAPK: mitogen-activated protein kinase
EMT: epithelial-to-mesenchymal transition.
