# Supplementary material for: Characterization of adipose-derived stem cells from subcutaneous and visceral adipose tissues and their function in breast cancer cells
Source: Oncotarget. 2015 Sep 30;6(33):34475–93. doi: 10.18632/oncotarget.5922 (PMC4741467; doi:10.18632/oncotarget.5922)
Supplement: Supplementary file 1 [file oncotarget-06-34475-s001.pdf]

Characterization of adipose-derived stem cells from subcutaneous and visceral adipose tissues and their function in breast cancer cells

Supplementary Material

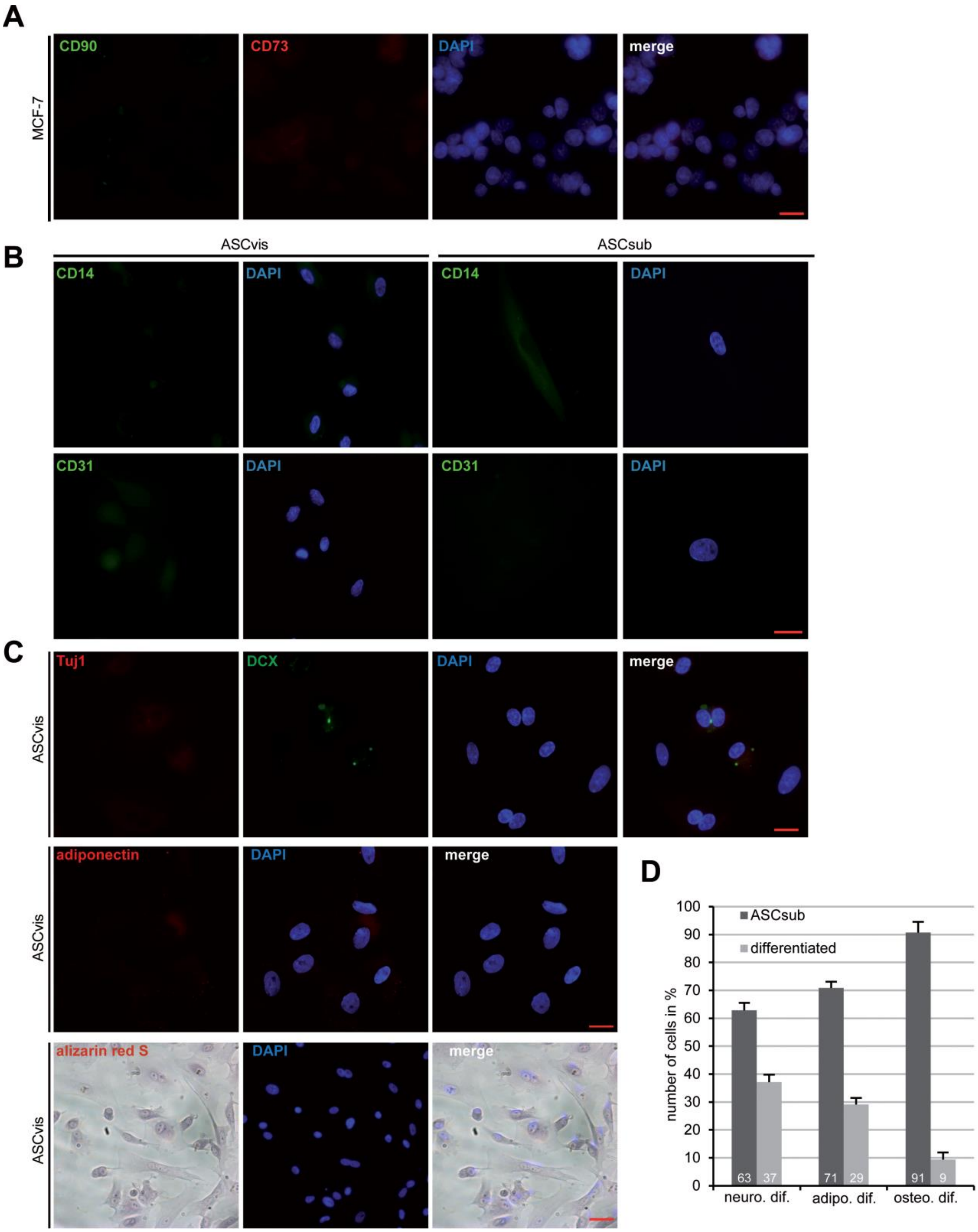

Figure S1: Negative CD markers of ASCs and differential potential of subcutaneous ASCs. (A) Immunofluorescence staining. MCF-7 cells were stained for CD90, CD73 and DNA for negative control. Representative is shown. Scale bar: 10 μm. (B) Immunofluorescence staining. Visceral ASCs (ASCvis) (left panel) and subcutaneous ASCs (ASCsub) (right panel) in passage 2 were stained for DNA, CD14, a cell surface marker typical for macrophages, neutrophils and dendritic cells, and CD31, characteristic for platelets, monocytes, neutrophils and endothelial cells. Representatives are shown. Scale bar: 10 μm. (C) Visceral ASCs (ASCvis) were stained for Tuj1, DCX, adiponectin, Alizarin Red S and DNA for negative control. Representatives are shown. Scale bar: 10 μm. (D) Quantification of the differentiation potential of subcutaneous ASCs by analyzing lineage-specific characteristic marker (n = 300 cells for each condition). The results are based on two independent experiments with ASCs obtained from two different donors and presented as mean ± SEM (n = 2).

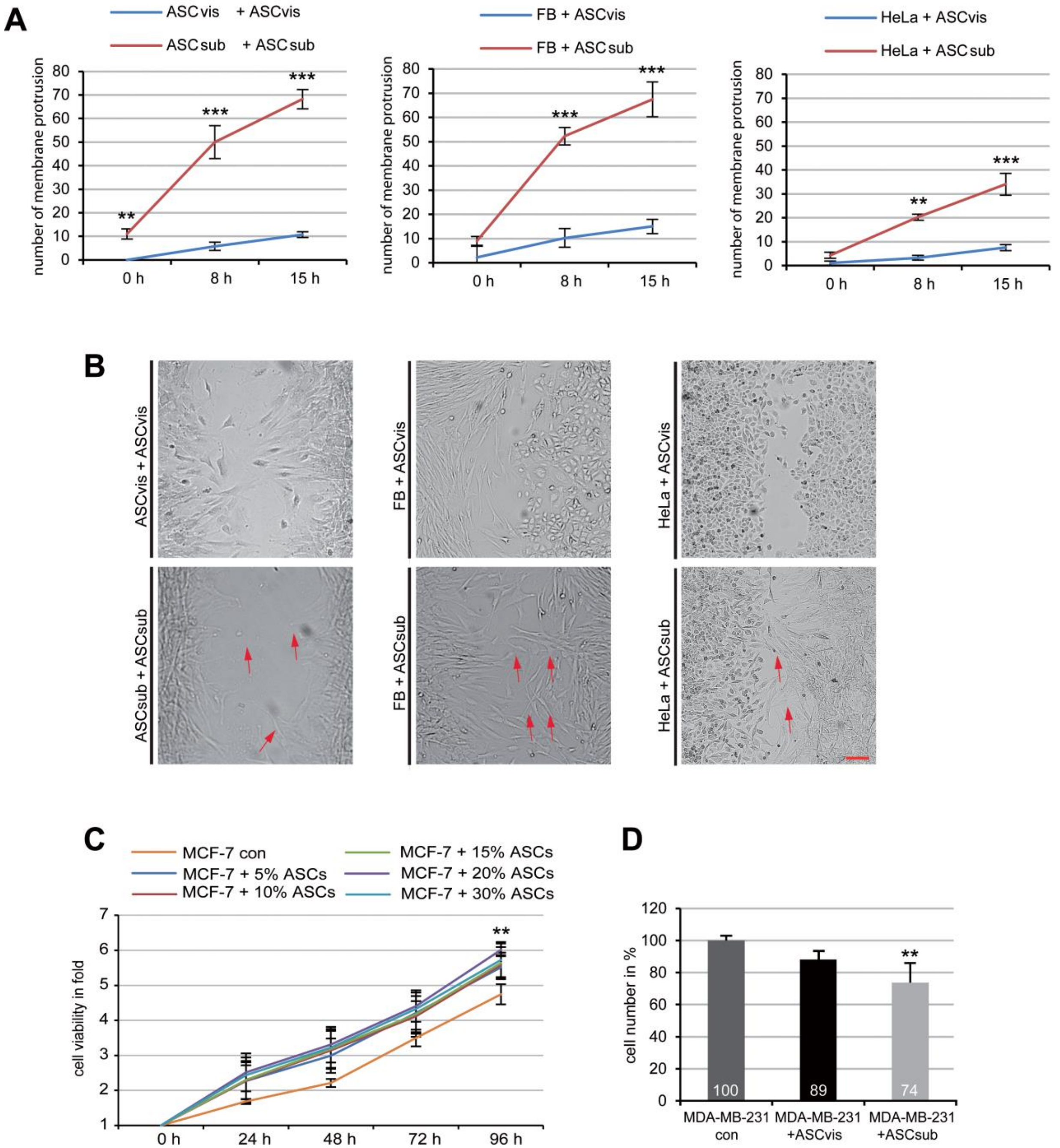

Figure S2: ASC homing ability. (A) Quantitative evaluation of membrane protraction of ASCs toward diverse cell lines as indicated. The data are based on three independent experiments with ASCs obtained from three different donors and presented as mean ± SEM. (B) Representatives of ASCs migrating toward themselves (left panels), fibroblasts (middle panels) and cervical carcinoma HeLa cells (right panels). Red arrows indicate protruded cell membranes of ASCs.

Scale bar: 250 μm. (C) To determine the optimal ASCs:MCF-7 ratio for further experiments, MCF-7 cells were co-cultured with visceral ASCs in different concentrations (5%, 10%, 15%, 20%, 30%) in 96-well plates for 0, 24, 48, 72, 96 h. Cell viability was measured via CellTiter-Blue® assay. The results are presented as mean ± SD (n = 3) and statistically analyzed. **$p < 0.01$. (D) Cell numbers were evaluated by flow cytometry in EGFP-H2B MDA-MB-231 cells cultured alone or in co-culture with visceral or subcutaneous ASCs for 5 days. The results are based from three independent experiments with ASCs from three different donors and presented as mean ± SEM. **$p < 0.01$.

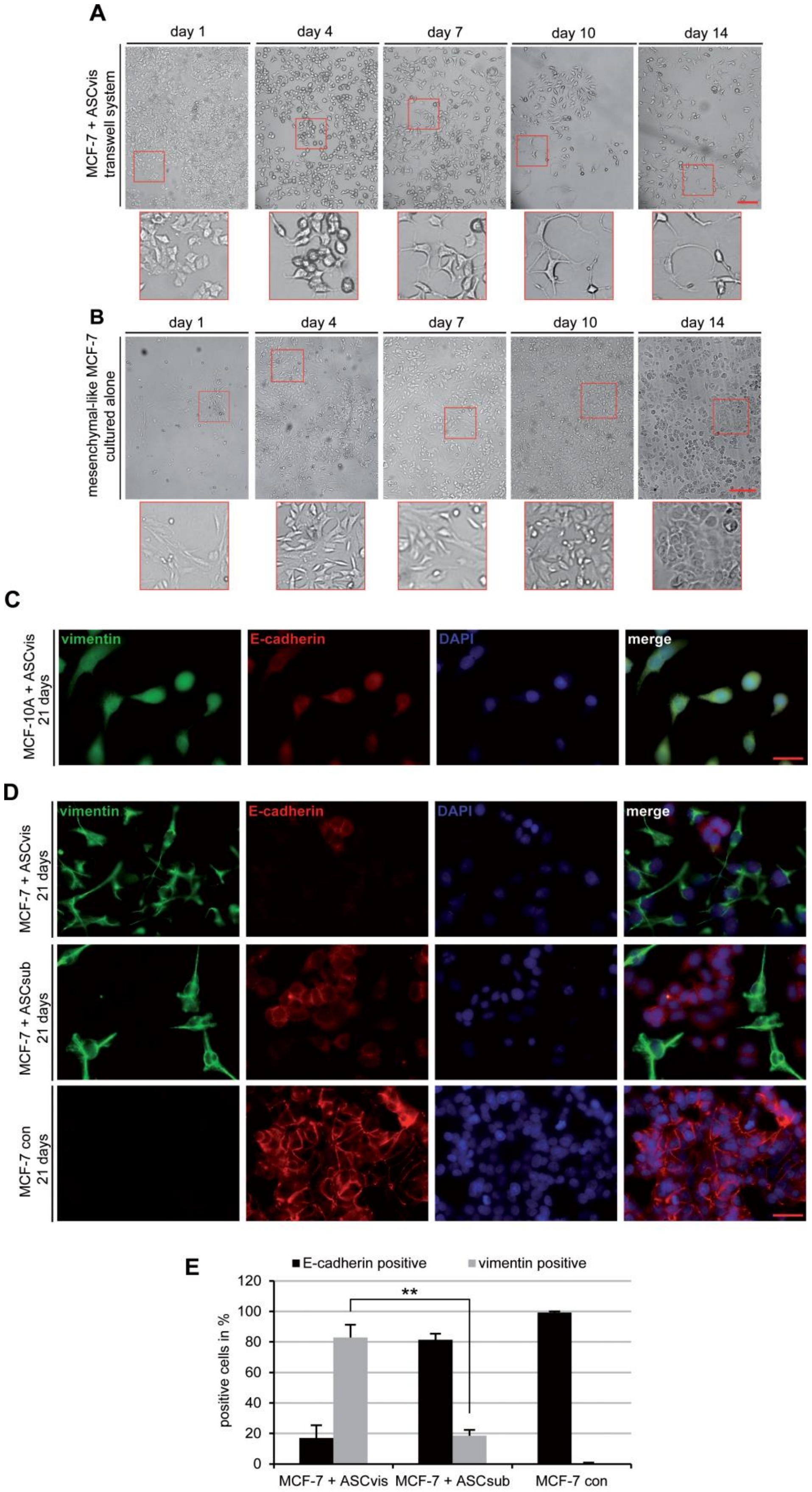
A
day 1
day 4
day 7
day 10
day 14
MCF-7 + ASCvis transwell system
B
mesenchymal-like MCF-7 cultured alone
C
MCF-10A + ASCvis 21 days
vimentin
E-cadherin
DAPI
merge
D
MCF-7 + ASCvis 21 days
MCF-7 + ASCsub 21 days
MCF-7 con 21 days
E
E-cadherin positive
vimentin positive
positive cells in %
**
0
20
40
60
80
100
120
MCF-7 + ASCvis
MCF-7 + ASCsub
MCF-7 con

Figure S3: EMT is a reversible process in MCF-7 and MCF-10A cells. (A) Bright-field images of MCF-7 indirectly co-cultured with visceral ASCs (ASCvis) in a transwell chamber system over indicated time points. Insets illustrate characteristic morphological changes during the transition of MCF-7 cells from epithelial-to-mesenchymal phenotype. (B) Bright-field images of mesenchymal-like MCF-7 cells released from co-culture for indicated time points. Insets depict the reversible morphological change of MET. (C) Immunofluorescence staining. Normal mammary epithelia MCF-10A cells were stained for E-cadherin, vimentin and DAPI after 21 days of indirect co-culture. Scale bar: 20 µm. (D) MCF-7 cells indirectly co-cultured with visceral ASCs or subcutaneous ASCs for 21 days were stained for the mesenchymal marker vimentin, the epithelial marker E-cadherin and DNA. Examples are shown. Scale bar: 50 µm. (E) Quantification of vimentin or E-cadherin positive MCF-7 cells following 21 days of indirect co-culture with visceral or subcutaneous ASCs. The results are presented as mean ± SD (n = 3). $^{**}p < 0.01$.

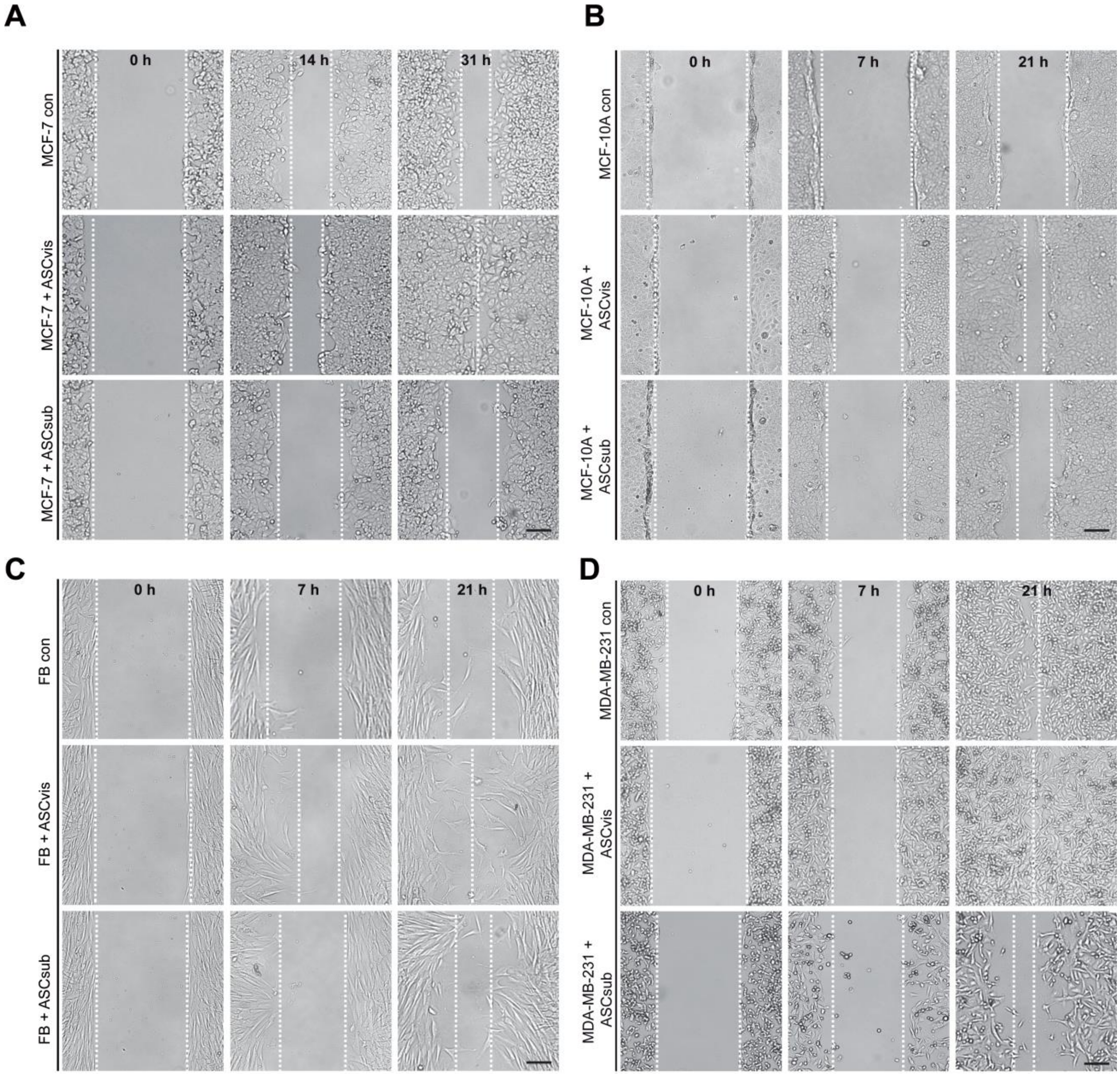

Figure S4: Visceral and subcutaneous ASCs influence the migration behavior of tumorigenic and non-tumorigenic cell lines. Wound healing/migration assays, as evaluated in Fig. 6A-D, were performed with MCF-7, MCF-10A, MDA-MB-231 and fibroblasts in the presence or absence of visceral ASCs (ASCvis) or subcutaneous ASCs (ASCsub) and pictures were taken at indicated time points to document the migration front. (A-D) Representatives are shown for MCF-7 (A), MCF-10A (B), fibroblasts (FB, C) and MDA-MB-231 cells (D). White dashed lines depict the migration front. Scale bar: 200 µm.
